# Supplementary material for: Hot spot prediction in protein-protein interactions by an ensemble system
Source: BMC Syst Biol. 2018 Dec 31;12(Suppl 9):132. doi: 10.1186/s12918-018-0665-8 (PMC6311905; doi:10.1186/s12918-018-0665-8)
Supplement: Supplementary file 2 — All relevant MATLAB code. A simple Matlab implement of our predictor. (ZIP 19,742 kb) [file 12918_2018_665_MOESM2_ESM.zip › Additional file 2/Read ASA/ASA website/dbMPIKT/1N8ZA.html]

 NetSurfP - Protein Surface Accessibility and Secondary Structure Predictions

  

|  |  |  |
| --- | --- | --- |
|  |  | NetSurfP - Protein Surface Accessibility and Secondary Structure Predictions  Technical University of Denmark |

  


---

```
# For publication of results, please cite:
# A generic method for assignment of reliability scores applied to solvent accessibility predictions.
# Bent Petersen, Thomas Nordahl Petersen, Pernille Andersen, Morten Nielsen and Claus Lundegaard
# BMC Structural Biology 2009, 9:51 doi:10.1186/1472-6807-9-51
#
# Column 1: Class assignment - B for buried or E for Exposed - Threshold: 25% exposure, but not based on RSA
# Column 2: Amino acid
# Column 3: Sequence name
# Column 4: Amino acid number
# Column 5: Relative Surface Accessibility - RSA
# Column 6: Absolute Surface Accessibility
# Column 7: Z-fit score for RSA prediction
# Column 8: Probability for Alpha-Helix
# Column 9: Probability for Beta-strand
# Column 10: Probability for Coil
E D  1N8ZA                  1    0.785 113.046   0.677   0.003   0.003   0.994
B I  1N8ZA                  2    0.190  35.076  -0.507   0.003   0.718   0.279
E Q  1N8ZA                  3    0.425  75.959   1.580   0.001   0.959   0.040
B M  1N8ZA                  4    0.115  23.112   1.118   0.001   0.959   0.040
E T  1N8ZA                  5    0.446  61.805   0.716   0.001   0.900   0.099
B Q  1N8ZA                  6    0.162  28.844   0.715   0.004   0.616   0.381
E S  1N8ZA                  7    0.330  38.676   0.262   0.004   0.138   0.858
E P  1N8ZA                  8    0.512  72.624  -1.027   0.018   0.019   0.964
E S  1N8ZA                  9    0.569  66.663  -1.089   0.018   0.088   0.893
E S  1N8ZA                 10    0.353  41.348  -1.297   0.022   0.359   0.619
B L  1N8ZA                 11    0.258  47.313  -0.290   0.021   0.756   0.223
E S  1N8ZA                 12    0.473  55.436  -0.800   0.018   0.846   0.136
B A  1N8ZA                 13    0.179  19.715  -0.015   0.023   0.655   0.322
E S  1N8ZA                 14    0.383  44.852   0.387   0.004   0.420   0.576
E V  1N8ZA                 15    0.494  75.943  -0.091   0.004   0.085   0.910
E G  1N8ZA                 16    0.680  53.485  -1.095   0.005   0.015   0.979
E D  1N8ZA                 17    0.536  77.223   0.270   0.004   0.085   0.910
E R  1N8ZA                 18    0.547 125.171   0.941   0.004   0.514   0.481
B V  1N8ZA                 19    0.071  10.851  -0.063   0.001   0.900   0.099
E T  1N8ZA                 20    0.424  58.753   1.508   0.001   0.959   0.040
B I  1N8ZA                 21    0.056  10.286   0.273   0.001   0.959   0.040
E T  1N8ZA                 22    0.351  48.711   0.781   0.001   0.900   0.099
B C  1N8ZA                 23    0.030   4.184   0.014   0.002   0.816   0.182
E R  1N8ZA                 24    0.419  95.974   0.977   0.001   0.900   0.099
B A  1N8ZA                 25    0.167  18.359  -1.138   0.002   0.816   0.182
E S  1N8ZA                 26    0.386  45.263  -0.957   0.004   0.616   0.381
E Q  1N8ZA                 27    0.573 102.391  -2.280   0.004   0.085   0.910
E D  1N8ZA                 28    0.514  73.995  -2.434   0.005   0.045   0.951
E V  1N8ZA                 29    0.509  78.310  -1.625   0.004   0.085   0.910
E N  1N8ZA                 30    0.460  67.359  -1.833   0.004   0.085   0.910
B T  1N8ZA                 31    0.220  30.486  -0.709   0.005   0.262   0.733
B A  1N8ZA                 32    0.155  17.037   0.195   0.003   0.718   0.279
B V  1N8ZA                 33    0.024   3.750   0.696   0.001   0.900   0.099
B A  1N8ZA                 34    0.063   6.921   0.525   0.001   0.959   0.040
B W  1N8ZA                 35    0.024   5.844   0.945   0.011   0.918   0.071
B Y  1N8ZA                 36    0.051  10.813   0.688   0.001   0.959   0.040
B Q  1N8ZA                 37    0.147  26.272   0.580   0.001   0.900   0.099
B Q  1N8ZA                 38    0.143  25.486   0.296   0.004   0.616   0.381
E K  1N8ZA                 39    0.437  89.850   1.435   0.004   0.085   0.910
E P  1N8ZA                 40    0.553  78.442  -0.603   0.005   0.015   0.979
E G  1N8ZA                 41    0.755  59.411  -2.079   0.003   0.003   0.994
E K  1N8ZA                 42    0.556 114.307   0.387   0.018   0.019   0.964
E A  1N8ZA                 43    0.349  38.471  -0.669   0.018   0.047   0.935
B P  1N8ZA                 44    0.141  19.965  -0.174   0.019   0.141   0.840
E K  1N8ZA                 45    0.385  79.256   0.482   0.022   0.552   0.426
B L  1N8ZA                 46    0.205  37.444   0.358   0.002   0.816   0.182
B L  1N8ZA                 47    0.083  15.179  -0.220   0.001   0.900   0.099
B I  1N8ZA                 48    0.026   4.755  -0.214   0.001   0.959   0.040
B Y  1N8ZA                 49    0.112  23.999  -0.149   0.001   0.959   0.040
B S  1N8ZA                 50    0.127  14.838  -0.732   0.001   0.900   0.099
B A  1N8ZA                 51    0.113  12.508  -1.029   0.022   0.552   0.426
B S  1N8ZA                 52    0.188  22.034  -1.659   0.020   0.205   0.775
E F  1N8ZA                 53    0.492  98.664  -1.684   0.019   0.141   0.840
E L  1N8ZA                 54    0.430  78.715  -0.679   0.018   0.047   0.935
E Y  1N8ZA                 55    0.634 135.443  -2.218   0.018   0.019   0.964
E S  1N8ZA                 56    0.691  81.044  -1.932   0.018   0.047   0.935
E G  1N8ZA                 57    0.558  43.930  -1.601   0.018   0.088   0.893
E V  1N8ZA                 58    0.307  47.217  -0.125   0.004   0.085   0.910
E P  1N8ZA                 59    0.504  71.475  -1.124   0.004   0.085   0.910
E S  1N8ZA                 60    0.465  54.486  -1.894   0.004   0.138   0.858
E R  1N8ZA                 61    0.311  71.105  -0.690   0.004   0.420   0.576
B F  1N8ZA                 62    0.095  19.026  -1.562   0.003   0.718   0.279
E S  1N8ZA                 63    0.297  34.855  -1.036   0.003   0.718   0.279
B G  1N8ZA                 64    0.259  20.360  -2.331   0.003   0.718   0.279
E S  1N8ZA                 65    0.418  48.978  -1.076   0.002   0.816   0.182
E R  1N8ZA                 66    0.411  94.188  -1.015   0.004   0.514   0.481
E S  1N8ZA                 67    0.533  62.432  -1.998   0.005   0.262   0.733
E G  1N8ZA                 68    0.621  48.841  -1.833   0.004   0.085   0.910
E T  1N8ZA                 69    0.379  52.567  -1.018   0.005   0.336   0.660
E D  1N8ZA                 70    0.400  57.683   0.032   0.002   0.816   0.182
B F  1N8ZA                 71    0.066  13.226  -0.405   0.001   0.900   0.099
B T  1N8ZA                 72    0.195  27.102  -0.108   0.001   0.900   0.099
B L  1N8ZA                 73    0.032   5.841   0.101   0.001   0.900   0.099
B T  1N8ZA                 74    0.237  32.927   0.205   0.001   0.900   0.099
B I  1N8ZA                 75    0.120  22.218  -0.408   0.002   0.816   0.182
E S  1N8ZA                 76    0.373  43.774   0.198   0.004   0.616   0.381
E S  1N8ZA                 77    0.420  49.165  -1.213   0.022   0.552   0.426
E L  1N8ZA                 78    0.370  67.802  -1.060   0.021   0.451   0.528
E Q  1N8ZA                 79    0.534  95.372  -0.067   0.022   0.359   0.619
E P  1N8ZA                 80    0.434  61.556  -0.385   0.022   0.359   0.619
E E  1N8ZA                 81    0.580 101.343  -1.293   0.005   0.336   0.660
E D  1N8ZA                 82    0.511  73.621  -0.810   0.004   0.420   0.576
E F  1N8ZA                 83    0.387  77.611  -0.626   0.004   0.514   0.481
B A  1N8ZA                 84    0.188  20.662  -1.434   0.004   0.514   0.481
E T  1N8ZA                 85    0.295  40.903  -0.034   0.002   0.816   0.182
B Y  1N8ZA                 86    0.074  15.750   0.191   0.001   0.900   0.099
B Y  1N8ZA                 87    0.106  22.716   0.569   0.001   0.900   0.099
B C  1N8ZA                 88    0.042   5.953  -0.296   0.002   0.816   0.182
B Q  1N8ZA                 89    0.190  33.934  -0.496   0.022   0.552   0.426
B Q  1N8ZA                 90    0.227  40.578  -0.899   0.020   0.205   0.775
E H  1N8ZA                 91    0.314  57.135  -1.563   0.019   0.141   0.840
E Y  1N8ZA                 92    0.398  85.138  -1.816   0.019   0.141   0.840
E T  1N8ZA                 93    0.538  74.607  -2.276   0.018   0.047   0.935
E T  1N8ZA                 94    0.307  42.567  -1.727   0.018   0.019   0.964
E P  1N8ZA                 95    0.334  47.324  -1.749   0.018   0.019   0.964
E P  1N8ZA                 96    0.402  57.072  -1.240   0.018   0.047   0.935
E T  1N8ZA                 97    0.295  40.930  -1.164   0.019   0.141   0.840
B F  1N8ZA                 98    0.172  34.420  -1.955   0.020   0.205   0.775
E G  1N8ZA                 99    0.364  28.678  -1.389   0.021   0.279   0.699
B Q  1N8ZA                100    0.285  50.883  -1.484   0.022   0.359   0.619
E G  1N8ZA                101    0.311  24.452  -1.253   0.022   0.359   0.619
E T  1N8ZA                102    0.347  48.198  -0.991   0.022   0.359   0.619
E K  1N8ZA                103    0.321  66.050  -1.220   0.022   0.359   0.619
E V  1N8ZA                104    0.380  58.375  -1.127   0.022   0.359   0.619
E E  1N8ZA                105    0.350  61.197  -0.826   0.022   0.359   0.619
E I  1N8ZA                106    0.314  58.146  -1.040   0.022   0.359   0.619
E K  1N8ZA                107    0.411  84.502  -0.998   0.022   0.359   0.619
E R  1N8ZA                108    0.409  93.753  -1.348   0.022   0.359   0.619
E T  1N8ZA                109    0.403  55.952  -1.234   0.021   0.279   0.699
E V  1N8ZA                110    0.393  60.343  -1.080   0.021   0.279   0.699
E A  1N8ZA                111    0.391  43.110  -1.039   0.022   0.359   0.619
E A  1N8ZA                112    0.404  44.499  -1.522   0.022   0.359   0.619
E P  1N8ZA                113    0.437  61.982  -1.156   0.022   0.359   0.619
E S  1N8ZA                114    0.401  47.044  -1.105   0.021   0.451   0.528
E V  1N8ZA                115    0.304  46.648  -1.243   0.021   0.451   0.528
E F  1N8ZA                116    0.392  78.735  -1.209   0.022   0.359   0.619
E I  1N8ZA                117    0.490  90.650  -1.841   0.021   0.279   0.699
E F  1N8ZA                118    0.427  85.779  -1.618   0.020   0.205   0.775
E P  1N8ZA                119    0.363  51.453  -1.701   0.056   0.142   0.802
E P  1N8ZA                120    0.499  70.851  -1.613   0.056   0.142   0.802
E S  1N8ZA                121    0.411  48.193  -1.524   0.056   0.142   0.802
E D  1N8ZA                122    0.406  58.548  -1.876   0.056   0.142   0.802
E E  1N8ZA                123    0.519  90.722  -2.235   0.056   0.142   0.802
E Q  1N8ZA                124    0.438  78.227  -1.965   0.052   0.084   0.864
E L  1N8ZA                125    0.419  76.737  -1.109   0.018   0.088   0.893
E K  1N8ZA                126    0.612 125.868  -1.650   0.018   0.019   0.964
E S  1N8ZA                127    0.699  81.970  -1.508   0.018   0.019   0.964
E G  1N8ZA                128    0.565  44.497  -1.441   0.004   0.085   0.910
E T  1N8ZA                129    0.419  58.171   0.224   0.005   0.336   0.660
B A  1N8ZA                130    0.074   8.111  -0.494   0.003   0.718   0.279
B S  1N8ZA                131    0.160  18.717   0.372   0.001   0.900   0.099
B V  1N8ZA                132    0.036   5.579  -0.034   0.001   0.900   0.099
B V  1N8ZA                133    0.044   6.809  -0.253   0.001   0.959   0.040
B C  1N8ZA                134    0.015   2.092   1.330   0.001   0.900   0.099
B L  1N8ZA                135    0.103  18.878   0.626   0.001   0.959   0.040
B L  1N8ZA                136    0.024   4.468   0.349   0.001   0.900   0.099
B N  1N8ZA                137    0.181  26.484   0.154   0.002   0.816   0.182
B N  1N8ZA                138    0.277  40.553  -1.759   0.004   0.616   0.381
B F  1N8ZA                139    0.250  50.095  -1.746   0.004   0.616   0.381
B Y  1N8ZA                140    0.243  51.865  -0.947   0.005   0.262   0.733
E P  1N8ZA                141    0.316  44.798  -1.820   0.004   0.085   0.910
E R  1N8ZA                142    0.605 138.499  -2.415   0.005   0.045   0.951
E E  1N8ZA                143    0.454  79.296  -1.200   0.004   0.197   0.799
B A  1N8ZA                144    0.142  15.593   0.013   0.003   0.718   0.279
B K  1N8ZA                145    0.278  57.102   0.257   0.001   0.900   0.099
B V  1N8ZA                146    0.043   6.594  -0.087   0.001   0.900   0.099
B Q  1N8ZA                147    0.172  30.683   0.460   0.011   0.918   0.071
B W  1N8ZA                148    0.043  10.221   0.246   0.011   0.918   0.071
B K  1N8ZA                149    0.208  42.703   0.892   0.001   0.900   0.099
E V  1N8ZA                150    0.271  41.714   0.252   0.002   0.816   0.182
E D  1N8ZA                151    0.544  78.448   0.031   0.020   0.205   0.775
E N  1N8ZA                152    0.634  92.862  -1.074   0.005   0.015   0.979
E A  1N8ZA                153    0.651  71.718  -0.983   0.005   0.045   0.951
E L  1N8ZA                154    0.663 121.469  -2.346   0.004   0.138   0.858
E Q  1N8ZA                155    0.482  86.049  -0.752   0.004   0.138   0.858
E S  1N8ZA                156    0.426  49.962  -1.371   0.005   0.045   0.951
E G  1N8ZA                157    0.606  47.661  -2.110   0.005   0.045   0.951
E N  1N8ZA                158    0.571  83.594  -1.728   0.004   0.197   0.799
E S  1N8ZA                159    0.416  48.732  -1.169   0.004   0.514   0.481
E Q  1N8ZA                160    0.357  63.832  -0.213   0.003   0.718   0.279
E E  1N8ZA                161    0.504  88.101  -1.421   0.022   0.552   0.426
E S  1N8ZA                162    0.458  53.631  -1.943   0.021   0.279   0.699
E V  1N8ZA                163    0.445  68.412  -1.333   0.021   0.279   0.699
E T  1N8ZA                164    0.325  45.119  -0.439   0.022   0.359   0.619
E E  1N8ZA                165    0.338  58.996  -1.559   0.022   0.359   0.619
E Q  1N8ZA                166    0.593 105.946  -1.572   0.021   0.451   0.528
E D  1N8ZA                167    0.474  68.361  -1.181   0.022   0.359   0.619
E S  1N8ZA                168    0.330  38.711  -1.894   0.004   0.197   0.799
E K  1N8ZA                169    0.643 132.245  -1.378   0.005   0.045   0.951
E D  1N8ZA                170    0.768 110.683  -2.486   0.005   0.015   0.979
E S  1N8ZA                171    0.471  55.166  -1.964   0.005   0.045   0.951
E T  1N8ZA                172    0.353  48.961  -0.580   0.004   0.420   0.576
B Y  1N8ZA                173    0.159  34.042  -1.511   0.004   0.616   0.381
B S  1N8ZA                174    0.130  15.213  -1.724   0.002   0.816   0.182
B L  1N8ZA                175    0.125  22.924  -1.088   0.001   0.900   0.099
B S  1N8ZA                176    0.197  23.053  -0.745   0.001   0.959   0.040
B S  1N8ZA                177    0.047   5.520  -1.321   0.001   0.959   0.040
B T  1N8ZA                178    0.185  25.604   0.207   0.001   0.959   0.040
B L  1N8ZA                179    0.041   7.599   0.070   0.001   0.900   0.099
E T  1N8ZA                180    0.303  41.957   0.688   0.001   0.900   0.099
B L  1N8ZA                181    0.074  13.549   0.607   0.002   0.816   0.182
E S  1N8ZA                182    0.357  41.829   0.984   0.021   0.451   0.528
E K  1N8ZA                183    0.431  88.636  -0.981   0.021   0.279   0.699
E A  1N8ZA                184    0.581  64.037  -1.163   0.020   0.205   0.775
E D  1N8ZA                185    0.541  77.915   0.855   0.020   0.205   0.775
B Y  1N8ZA                186    0.186  39.748  -0.965   0.018   0.088   0.893
E E  1N8ZA                187    0.663 115.844   0.490   0.005   0.045   0.951
E K  1N8ZA                188    0.668 137.449   0.568   0.005   0.045   0.951
E H  1N8ZA                189    0.631 114.761  -0.282   0.005   0.045   0.951
E K  1N8ZA                190    0.477  98.139   0.238   0.005   0.336   0.660
E V  1N8ZA                191    0.443  68.135   1.633   0.002   0.816   0.182
B Y  1N8ZA                192    0.070  14.874   1.172   0.001   0.959   0.040
E A  1N8ZA                193    0.289  31.837   1.801   0.001   0.959   0.040
B C  1N8ZA                194    0.027   3.847   0.625   0.001   0.959   0.040
B E  1N8ZA                195    0.243  42.487   1.331   0.001   0.959   0.040
B V  1N8ZA                196    0.086  13.234   0.423   0.001   0.900   0.099
E T  1N8ZA                197    0.409  56.770   0.675   0.001   0.900   0.099
B H  1N8ZA                198    0.141  25.721   0.344   0.002   0.816   0.182
E Q  1N8ZA                199    0.618 110.428  -0.121   0.005   0.262   0.733
E G  1N8ZA                200    0.617  48.582  -1.765   0.005   0.045   0.951
E L  1N8ZA                201    0.382  69.871  -0.813   0.005   0.045   0.951
E S  1N8ZA                202    0.653  76.520  -0.112   0.005   0.045   0.951
E S  1N8ZA                203    0.551  64.577   0.314   0.018   0.088   0.893
E P  1N8ZA                204    0.432  61.287   0.109   0.021   0.279   0.699
B V  1N8ZA                205    0.188  28.896  -0.484   0.004   0.616   0.381
E T  1N8ZA                206    0.558  77.395   0.199   0.002   0.816   0.182
E K  1N8ZA                207    0.274  56.444   1.189   0.001   0.900   0.099
E S  1N8ZA                208    0.486  56.924   0.168   0.001   0.900   0.099
B F  1N8ZA                209    0.113  22.759   0.868   0.002   0.816   0.182
E N  1N8ZA                210    0.497  72.819   1.552   0.004   0.616   0.381
E R  1N8ZA                211    0.473 108.386   0.557   0.020   0.205   0.775
E G  1N8ZA                212    0.655  51.509  -1.114   0.019   0.141   0.840
E E  1N8ZA                213    0.634 110.725   1.385   0.018   0.088   0.893
E C  1N8ZA                214    0.490  68.824  -1.314   0.003   0.003   0.994

Explain the output.  Go back.
```

---
